# Supplementary material for: Identification of photoperiod-induced specific miRNAs in the adrenal glands of Sunite sheep (Ovis aries)
Source: Front Vet Sci. 2022 Jul 22;9:888207. doi: 10.3389/fvets.2022.888207 (PMC9354845; doi:10.3389/fvets.2022.888207)
Supplement: Supplementary file 1 [file Data_Sheet_1.ZIP › Supplementary file/miRDeep2 file.zip!/miRDeep2 file/Novel_Structure/SP42A1/Novel_554.pdf]

Provisional ID	:	NC_019464.2_11231
Score total	:	1192.7
Score for star read(s)	:	3.9
Score for read counts	:	1185
Score for mfe	:	2.2
Score for randfold	:	1.6
Score for cons. seed	:	
Total read count	:	2336
Mature read count	:	2316
Loop read count	:	0
Star read count	:	20

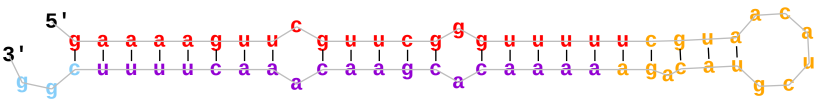

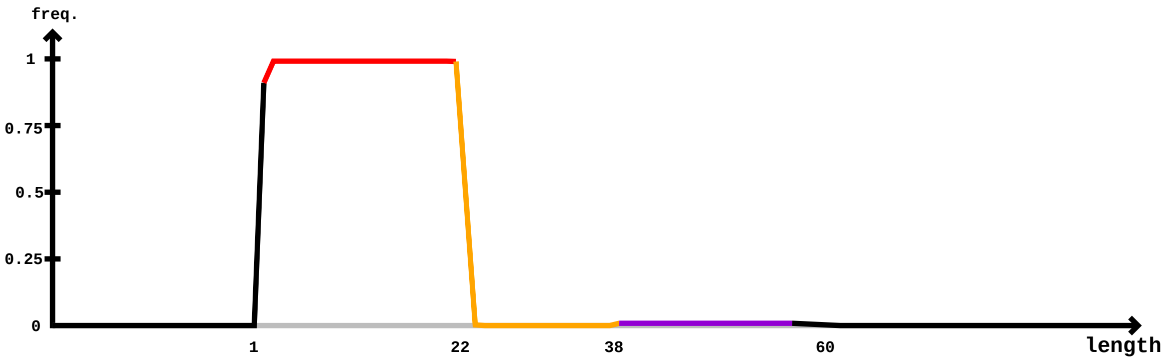

Mature **Star**

```
5'- auuaucauauuggguuggcugaaaaguucguucggguuuuucguaacaucguacagaaaaacacgaacaaacuuuucggccaacccaguauuucauucaucugcguuuucc   -3'   obs
    auuaucauauuggguuggcugaaaaguucguucggguuuuucguaacaucguacagaaaaacacgaacaaacuuuucggccaacccaguauuucauucaucugcguuuucc         exp
    ......((((((((((((((((((((((.(((((.((((((((((......))).))))))).))))).)))))))))))))))))))))))....................   reads   mm   sample
    ....................gaaaaguucguucggguuuuu......................................................................   2125    0    seq
    ....................Aaaaaguucguucggguuuuuc.....................................................................   1       1    seq
    .....................aaaaguucguucggguuuu.......................................................................   3       0    seq
    .....................aaaaguucguucggguuuuu......................................................................   99      0    seq
    .....................aaaaguucguucggguuuuC......................................................................   9       1    seq
    .....................aaaaguucguucggguuuuuc.....................................................................   73      0    seq
    .....................aaaaguucguucggguuuuucU....................................................................   6       1    seq
    .........................................................aaaacacgaacaaacuuuu...................................   20      0    seq
```
